# Probing oral anticoagulation in patients with atrial high rate episodes: Rationale and design of the Non–vitamin K antagonist Oral anticoagulants in patients with Atrial High rate episodes (NOAH–AFNET 6) trial

**DOI:** 10.1016/j.ahj.2017.04.015

**Published:** 2017-08

**Authors:** Paulus Kirchhof, Benjamin F. Blank, Melanie Calvert, A. John Camm, Gregory Chlouverakis, Hans-Christoph Diener, Andreas Goette, Andrea Huening, Gregory Y.H. Lip, Emmanuel Simantirakis, Panos Vardas

**Affiliations:** a Institute of Cardiovascular Sciences, University of Birmingham, Birmingham, United Kingdom; b Department of Cardiology, SWBH NHS trust, Birmingham, United Kingdom; c Department of Cardiology, UHB NHS Foundation trust, Birmingham, United Kingdom; d AFNET (Kompetenznetz Vorhofflimmern e.V.), Muenster, Germany; e Centre for Patient Reported Outcomes Research, University of Birmingham, Edgbaston, Birmingham, United Kingdom; f Institute of Applied Health Research, University of Birmingham, Edgbaston, Birmingham, United Kingdom; g Cardiovascular and Cell Sciences Research Institute, St George's, University of London, and Imperial College, London, United Kingdom; h Biostatistics Lab, School of Medicine, University of Crete, Crete, Greece; i Department of Neurology, University Hospital Essen, Essen, Germany; j Department of Cardiology and Intensive Care Medicine, St Vincenz-Hospital Paderborn, Paderborn, Germany; k The Clinical Research Institute, Munich, Germany; l Aalborg Thrombosis Research Unit, Department of Clinical Medicine, Aalborg University, Aalborg, Denmark; m Department of Cardiology, Heraklion University Hospital, Crete, Greece

## Abstract

Oral anticoagulation prevents ischemic strokes in patients with atrial fibrillation (AF). Early detection of AF and subsequent initiation of oral anticoagulation help to prevent strokes in AF patients. Implanted cardiac pacemakers and defibrillators allow seamless detection of atrial high rate episodes (AHRE), but the best antithrombotic therapy in patients with AHRE is not known.

**Rationale:**

Stroke risk is higher in pacemaker patients with AHRE than in those without, but the available data also show that stroke risk in patients with AHRE is lower than in patients with AF. Furthermore, only a minority of patients with AHRE will develop AF, many strokes occur without a temporal relation to AHRE, and AHRE can reflect other arrhythmias than AF or artifacts. An adequately powered controlled trial of oral anticoagulation in patients with AHRE is needed.

**Design:**

The **N**on–vitamin K antagonist **O**ral anticoagulants in patients with **A**trial **H**igh rate episodes (NOAH–AFNET 6 ) trial tests whether oral anticoagulation with edoxaban is superior to prevent the primary efficacy outcome of stroke or cardiovascular death compared with aspirin or no antithrombotic therapy based on evidence-based indications. The primary safety outcome will be major bleeding. NOAH–AFNET 6 will randomize 3,400 patients with AHRE, but without documented AF, aged ≥65 years with at least 1 other stroke risk factor, to oral anticoagulation therapy (edoxaban) or no anticoagulation. All patients will be followed until the end of this investigator-driven, prospective, parallel-group, randomized, event-driven, double-blind, multicenter phase IIIb trial. Patients will be censored when they develop AF and offered open-label anticoagulation. The sponsor is the Atrial Fibrillation NETwork (AFNET). The trial is supported by the DZHK (German Centre for Cardiovascular Research), the BMBF (German Ministry of Education and Research), and Daiichi Sankyo Europe.

**Conclusion:**

NOAH–AFNET 6 will provide robust information on the effect of oral anticoagulation in patients with atrial high rate episodes detected by implanted devices.

## Graphical abstract

**Figure:**
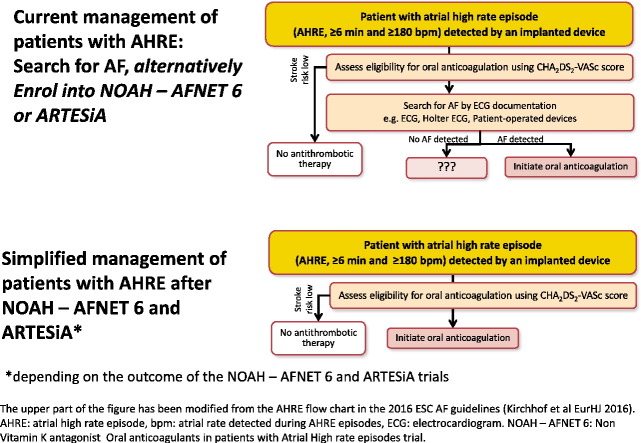

## Background and rationale

Atrial fibrillation (AF) is a common cause of stroke, especially ischemic stroke. Unlike strokes of other major etiologies, which can be prevented by antiplatelet therapy, stroke prevention in patients with AF requires oral anticoagulation.1., 2., 3. Recently, 4 non–vitamin K antagonist oral anticoagulants (NOACs) have been introduced into clinical practice as alternatives to vitamin K antagonist (VKA) therapy.4., 5., 6., 7., 8. NOACs provide similar stroke prevention efficacy and are at least as safe as VKA,8., 9. with less intracranial hemorrhage and a 10% reduction in mortality in the pivotal trials.^10^

So far, all available data that demonstrate a beneficial effect of oral anticoagulation for stroke prevention have been collected in populations with AF documented by conventional electrocardiogram (ECG).2., 3. Studies in other populations in the absence of AF, for example, heart failure or survivors of a stroke, had overall neutral outcomes, where slight reductions in stroke were counterbalanced by increased bleeding.11., 12. Therefore, antiplatelet therapy is recommended for secondary prevention of ischemic strokes in patients without AF or other cardioembolic cause of stroke.

Many AF episodes remain undiagnosed.2., 3. Often, a potentially preventable stroke is the first clinical manifestation of hitherto undetected AF (silent AF). In fact, around 5% of unselected patients presenting with an acute stroke suffer from silent AF that is detected by a simple ECG upon admission.13., 14. Published and ongoing studies suggest that systematic ECG screening using patient-operated devices15., 16., 17. or “opportunistic screening” in those presenting to a health care professional^18^ can detect silent AF. This is in line with predictions made from trial data sets.^19^ Systematic ECG monitoring in stroke survivors detects silent paroxysmal AF in approximately 5% of unselected stroke patients13., 20. and in up to 20% when long-term ECG monitoring is applied to patients with cryptogenic stroke.21., 22., 23. Even these prolonged ECG monitoring techniques will miss silent AF in many patients.^19^ Continuous monitoring of atrial rhythm could close this diagnostic gap,^19^ requiring implanted devices and automated or semiautomated analysis.21., 22., 23.

Most modern pacemakers, defibrillators, and cardiac resynchronization devices provide automated algorithms alerting to the occurrence of atrial high rate episodes, also called *subclinical atrial fibrillation* or, more appropriately, *device-detected atrial high rate episodes* (AHRE).2., 24., 25. Data from large prospectively followed patient cohorts have demonstrated that the presence of AHRE increases stroke risk.24., 26., 27. Only a minority of patients with AHRE (estimated at 13%-16% over 2.5 years) will develop AF.24., 28.

The absolute stroke rates are lower in patients with AHRE when compared with stroke rates in patients with clinically diagnosed AF.24., 26., 27., 29., 30. For example, the TRENDS cohort, collecting information on patients with long AHRE episodes, reported a stroke rate of 1.1%-2.2% per year.^30^ One of the largest cohorts, SOS, reported AHRE and stroke rates in >10,000 unselected device patients (mean age 70 years, more than 50% with additional stroke risk factors, 80% not anticoagulated).^27^ AHRE were found in 43% of all patients. The annual rate of stroke or transient ischemic attack was between 0.32% and 0.67% per year. Stroke rates of around 1.7% per year were reported in patients with AHRE enrolled into the ASSERT^24^ and IMPACT trials.^31^ Higher stroke rates were reported in other smaller and earlier series (Table II). The SOS data set did not show a clear increase in patients with higher AHRE burden (Figure 3 in Boriani et al^27^). Other analyses in smaller populations have suggested that longer AHRE episodes (eg, those lasting >24 hours) are associated with a higher stroke risk.32., 33. Furthermore, automated detection algorithms of AHRE by implanted devices have a good sensitivity, but their specificity may not be optimal, especially for short episodes,^34^ to constantly distinguish AF from other arrhythmias or artifacts.25., 35. As an illustrative example, 15% (19/130) patients without AF on a simultaneously recorded Holter ECG were classified as having AF on an implanted loop recorder.^34^ In summary, stroke rates in patients with AHRE are often close to the threshold for a net clinical benefit of NOAC therapy^2^ and appear lower than the stroke rates found in patients with paroxysmal AF detected by ECG.5., 36., 37.

Also, only a minority of ischemic strokes in pacemaker patients with AHRE occur in relation to the time of an AHRE episode.24., 27., 30., 31., 38., 39., 40. Thus, there are important uncertainties regarding the prognostic and therapeutic implications of device-detected AHRE (Text box). Reacting to these uncertainties, several groups have proposed to evaluate intermittent oral anticoagulation in AHRE patients.31., 41. One prior study (IMPACT) did not find a benefit of intermittent oral anticoagulation compared with no anticoagulation in 2,718 AHRE patients.^31^ In light of the bleeding complications associated with oral anticoagulation,42., 43., 44., 45. patients with AHRE should currently only be anticoagulated once AF has been documented by ECG,46., 47., 48. a resource-intensive and time-consuming process.2., 25. A sufficiently powered randomized trial of continuous oral anticoagulation compared with no anticoagulation is needed to inform optimal stroke prevention in AHRE patients.

**Text box**

Current uncertainties relating to the detection of AHRE and to the management of patients with AHRE

1. The prognostic impact (eg, increase in stroke risk) of rare and short episodes of atrial fibrillation or other atrial arrhythmias documented on CIED logs, often only a few hours per year, is probably lower than that of ECG-diagnosed atrial fibrillation
2. Some AHRE reflect other atrial arrhythmias than atrial fibrillation that do not require oral anticoagulation for stroke prevention
3. The timing of AHRE is not closely related to strokes observed in patients with AHRE, suggesting that other mechanisms than cardioembolic stroke underlie these events
4. AHRE episodes may be artifacts, especially those of shorter duration.

Alt-text: Image 2

### Design of the Non–vitamin K antagonist Oral anticoagulants in patients with Atrial High rate episodes trial

#### Hypothesis

The Non–vitamin K antagonist Oral anticoagulants in patients with Atrial High rate episodes (NOAH–AFNET 6) trial tests the hypothesis that oral anticoagulation with the NOAC edoxaban is superior to current therapy (antiplatelet therapy or no therapy depending on cardiovascular risk) to prevent stroke, systemic embolism, or cardiovascular death in patients with AHRE, but without AF, and with at least 2 stroke risk factors. We will also assess whether the intervention improves quality of life and maintains cognitive function. NOAH–AFNET 6 is registered at clinicaltrials.gov (NCT02618577), EudraCT (2015-003997-33), and ISRCTN (17309850). The protocol was developed in accordance with the Standard Protocol Items for Randomized Trials statement^49^ and guidance from the International Society for Quality of Life Research Best Practice task force.50., 51. No funding other than the sources mentioned in the acknowledgment was used to support this work. The authors and the study sponsor, AFNET, are solely responsible for the design and conduct of this study, all study analyses, the drafting and editing of the paper, and its final contents. Further information can be obtained from the trial Web site: www.noah.af-net.eu.

### Patient population

NOAH–AFNET 6 will enroll patients with AHRE but without diagnosed AF or another accepted indication for oral anticoagulation. Patients in the trial would qualify for oral anticoagulation if they had diagnosed AF, approximated by an age of 65 years or more and presence of at least 1 additional clinical stroke risk factor.^2^ The trial has broad inclusion and exclusion criteria in line with a simple trial that is close to clinical practice (see Table I for details). It has been reported that approximately 1 of 7 (13%-16%) of patients with device-detected AHRE will develop AF over a mean follow-up of 2.5 years.24., 26. Patients will undergo ECG in 6-month intervals (at baseline and during follow-up). Patients will be censored at the time of developing AF and taken off study medication, as they have an indication for oral anticoagulation. All patients will be followed up until the end of the trial. To date, 118 sites have been initiated in 11 countries. One hundred thirty-one patients have been randomized so far.

**Table I:** Inclusion and exclusion criteria of the NOAH–AFNET 6 trial

Inclusion criteria	Exclusion criteria
I1. Pacemaker or defibrillator implanted for any reason with feature of detection of AHRE, implanted at least 2 m prior to randomization	E1. Any disease that limits life expectancy to less than 1 y
I2. AHRE detection feature activated	E2. Participation in another controlled clinical trial, either within the past 2 months or still ongoing
I3. AHRE (≥180 beats/min atrial rate and ≥6-min duration) documented by the implanted device via its atrial lead and stored digitally	E3. Previous participation in the present trial NOAH–AFNET 6
I4. Age ≥65 y	E4. Drug abuse or clinically manifest alcohol abuse
I5. In addition, at least 1 of the following cardiovascular conditions - Age ≥75 y, - Heart failure (clinically overt or LVEF <45%), - Arterial hypertension (long-term treatment for hypertension, estimated need for continuous antihypertensive therapy, or resting blood pressure >145/90 mm Hg), - Diabetes mellitus, - Prior stroke or TIA, or - Vascular disease (peripheral, carotid/cerebral, or aortic plaques on TEE).	E5. Any history of AF or atrial flutter or presence of AF at baseline 12-lead ECG
I6. Provision of signed informed consent	E6. Indication for oral anticoagulation (eg, deep venous thrombosis)
	E7. Contraindication for oral anticoagulation in general
	E8. Contraindication for edoxaban as stated in the current SmPC
	E9. Indication for long-term antiplatelet therapy other than acetylsalicylic acid, especially DAPT
	E10. Acute coronary syndrome, coronary revascularization (PCI or bypass surgery), or overt stroke within 30 d prior to randomization
	E11. End stage renal disease (CrCl <15 mL/min)

The inclusion criteria I4 and I5 closely resemble the criteria of a CHA2DS2-VASc score of ≥2 in patients with diagnosed AF. Patients with a transient requirement for dual antiplatelet therapy (DAPT, eg, after receiving a stent) will be eligible when the need for DAPT is no longer present.

*LVEF*, Left ventricular ejection fraction; *TIA*, transient ischemic attack; *TEE*, transesophageal echocardiogram; *SmPC*, summary of product characteristics; *DAPT*, dual-antiplatelet therapy; *PCI*, percutaneous coronary intervention; *CrCL*, creatinine clearance.

### Trial organization

NOAH–AFNET 6 is an investigator-initiated, prospective, parallel-group, double-blind, randomized, multicenter phase IIIb trial (Figure). Study patients will be randomized to 1 of 2 parallel groups in a 1:1 design, designated as *NOAC* and *Usual Care*. Randomization will be stratified by indication for use of antiplatelet therapy as assessed by the responsible site investigator at the time of randomization. Patients randomized to the NOAC group will receive edoxaban in the therapeutic dose approved for stroke prevention in AF, that is, 60 mg od with a reduction of dose to 30 mg od in patients with impaired renal function, low body weight, or receiving certain glycoprotein P inhibitors. Patients randomized to usual care will receive either placebo or aspirin depending on established indications for aspirin therapy. NOAH–AFNET 6 is a joint project of the Atrial Fibrillation NETwork (AFNET) and of the European Society of Cardiology. AFNET is sponsor of the trial. The study is led by an academic steering committee and overseen by a data safety and monitoring board. The trial will be conducted with the help of a clinical research organization, the Clinical Research Institute based in Munich.

**Figure:**
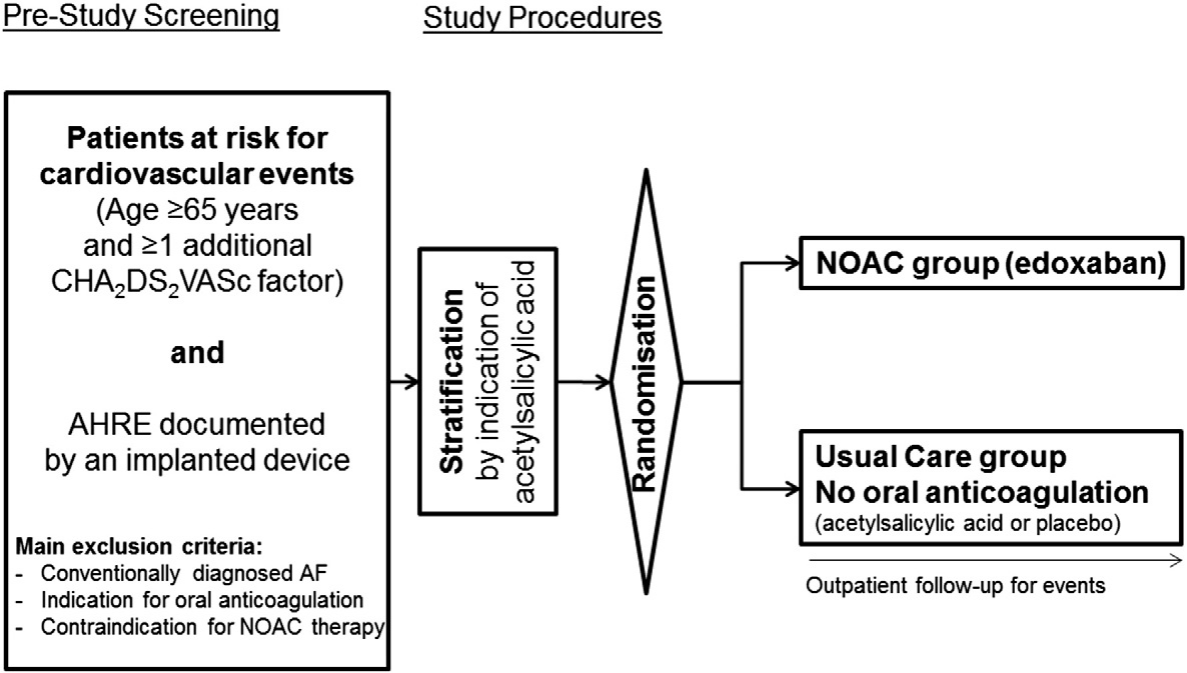
Flowchart of NOAH–AFNET 6. In the setting of NOAH–AFNET 6, edoxaban 60 mg od is the NOAC of choice. The dose will be reduced to 30 mg od (in accordance with the label for AF) in patients with one of the following characteristics: (1) impaired renal function (creatinine clearance 15-50 mL/min); (2) low body weight (≤60 kg); or (3) patients receiving the glycoprotein P inhibitors ketoconazole, cyclosporine, erythromycin, or dronedarone. In a double-blind design, established indications in the inclusion criteria for antiplatelet therapy will guide the use of blind aspirin or blind placebo for the patients not receiving anticoagulation. Patients randomized to NOAC will not receive aspirin in addition. All patients will be followed until the end of the trial for events. ECGs will be recorded at baseline and in 6-month intervals during follow-up.

NOAH–AFNET 6 is supported by Daiichi Sankyo Europe; the DZHK (German Centre for Cardiovascular Research); and the BMBF (German Ministry of Education and Research, FKZ 81x2800110).

#### Outcome parameters

The primary outcome parameter of NOAH–AFNET 6 is a composite of stroke, systemic embolism, or cardiovascular death, measured as the time from randomization to the first occurrence of either of these events. All events will be centrally adjudicated by an independent committee. Secondary outcome parameters include components of the primary outcome, all-cause death, major bleeding events according to the ISTH definitions, changes in quality of life assessed using the EQ-5D and the Karnofsky scale,^52^ patient satisfaction assessed by the modified European Heart Rhythm Association Symptoms (mEHRA) score2., 53. and PACT-Q,51., 52. and cognitive function assessed by the Montreal Cognitive Assessment (MoCA) at 12 and 24 months compared with baseline. Cost-effectiveness and health resource utilization will be assessed. All AHRE episodes will be reanalyzed by an independent core laboratory based at Maastricht University to verify whether AHRE data uploaded by trial sites fulfill the requirements of the protocol as inclusion criteria. The core laboratory also performs quality control and provides feedback to trial sites regarding their uploaded AHRE data if necessary. Prespecified exploratory analyses will include markers identifying patients at high stroke risk, for example, by duration or pattern of AHRE episodes, or using blood/ECG analyses. All patients will be followed up until the end of the trial.

#### Estimation of the event rate and effect size for the primary outcome in the NOAH–AFNET 6 population

To arrive at a reliable estimate of the control group event rate in the NOAH–AFNET 6 patient population, stroke/systemic embolism and cardiovascular death rates in patients with AHRE in published series were compiled (Table II). Based on this analysis, considering stroke and death rates in nonanticoagulated patients with paroxysmal AF2., 5., 36., 37. and accounting for the variability in stroke rate estimates in published series of patients with AHRE (Table II), an annual stroke rate of 1.9% and an annual cardiovascular death rate of 3.3% are expected, or a rate of the primary outcome of 5.3% per year. For the purpose of NOAH–AFNET 6, patients randomized to edoxaban are expected to have a two-thirds lower rate of stroke or TIA5., 54. and a 10% reduction in cardiovascular death. The effect estimate for stroke prevention is based on the effect size observed for stroke prevention with VKA (warfarin) or a NOAC (apixaban) compared with acetylsalicylic acid in patients with AF,5., 36. assuming that edoxaban is at least as effective as dose-adjusted warfarin in preventing strokes in AHRE patients. The effect size for reduction in cardiovascular death is the point estimate of reduction in cardiovascular deaths observed in the 4 NOAC trials compared with warfarin.^10^ This conservative estimate is based on the assumption that the mortality benefit of edoxaban compared with no anticoagulation in NOAH–AFNET 6 is not bigger than the mortality benefit of edoxaban compared with dose-adjusted warfarin observed in ENGAGE-TIMI 48^8^ and in the other 3 NOAC trials.^10^ These assumptions result in an estimated event rate of 0.62% per year for stroke and 3% for cardiovascular death. The expected event rate in the edoxaban group in NOAH–AFNET 6 (3.62% per year) is very similar to the observed rate of stroke or cardiovascular death in those patients enrolled in ENGAGE and randomized to edoxaban who match the NOAH–AFNET 6 inclusion criteria (3.45% per year, data on file).

**Table II:** Annualized rate of stroke and cardiovascular death in patients with device-detected AHRE

Study/population	Stroke	Cardiovascular death	Sum
ASSERT,^24^ 261 pts, 2.5-y FU	1.7	2.9	4.5
MOST,^26^ 160 pts, 2.25-y FU†	5	4.2	9.2
AT500,^55^ 725 pts, 1.8-y FU†	3.6		
Botto et al,^56^ 223 pts, 1-y FU	3.2		
TRENDS,^30^ 1.4-y FU†	1.2		
SOS,^27^ 10,106 pts, 2.2-y FU⁎	0.4-0.8		

Event rates are given as percentage per year, split by stroke and cardiovascular death. The sum of these 2 components provides a first estimate of the primary outcome rate. All events are rounded to 1 decimal percentage point.

The table lists event rates that were available at the time of designing NOAH–AFNET 6.

⁎ SOS included approximately 20% of anticoagulated patients. Death and bleeding event rates are not reported.

† MOST, TRENDS, and AT500 included patients with atrial fibrillation diagnosed by ECG, thereby probably enriching for patients at higher stroke risk.

The potential benefit of oral anticoagulation in the NOAH–AFNET 6 population will have to be weighed against the bleeding risk associated with long-term NOAC therapy (Table III). It is worthwhile to note the IMPACT trial in this context: IMPACT did not find a benefit of intermittent anticoagulation, initiated at the time of device-detected AHRE and continued for 30 to 90 days after documentation of the last AHRE event, to usual care (no anticoagulation) in 2,718 patients with AHRE and a median CHA2DS2-VASc score of 4.^31^ IMPACT observed 22 ischemic strokes and 41 major bleeding events, illustrating the equipoise for anticoagulation in patients with device-detected AHRE and stroke risk factors.

**Table III:** Comparison of published annualized ischemic and bleeding event rates in populations with diagnosed AF that otherwise resemble the projected NOAH–AFNET 6 population

	Stroke, systemic embolism, or cardiovascular death	Fatal bleeding	Hemorrhagic stroke plus other intracranial hemorrhage	Major extracranial hemorrhage
ENGAGE-TIMI 48 edoxaban 60 mg od dosing regime^8^	3.85	0.20	0.39	2.65
AVERROES aspirin arm^5^	6.40	0.20	0.50	1.2⁎
BAFTA aspirin arm^36^	8.10	0.10	0.50	2.00

The chart shows major efficacy (stroke or cardiovascular death, red) and safety events (fatal bleeding, hemorrhagic stroke or intracranial hemorrhage, major extracranial bleeding) in controlled trials comparing anticoagulation with aspirin and in the ENGAGE-TIMI48 trial evaluating edoxaban.

⁎ For the AVERROES acetylsalicylic acid group, only the AVERROES major bleeds are reported, although some of the “clinically relevant nonmajor bleeds” in AVERROES would have been classified as “major” in BAFTA and ENGAGE.

#### Sample size

Three thousand four hundred patients will be randomized. Computation of sample size is based on a hazard ratio of 0.68, accrual period of 27 months, and minimum follow-up of 12 months. Specifically, it assumes annual hazard rates of 5.3% for the standard treatment group versus 3.62% for the NOAC group. This is equivalent to median event-free survival of 13 years for the standard treatment group versus 19 years for the NOAC group. This effect size is considered realistic in view of the effect of continuous oral anticoagulation in patients with AF. The minimum number of events that need to be observed to detect the aforementioned difference as significant at the 2-sided 5% level, with 80% power, is 222. Given the accrual and follow-up periods, at least 1,260 patients per group are needed for that number of events during the trial duration. The hazard of developing overt AF or of dropping out of the trial for other reasons is assumed to be at 0.287 throughout the trial, equivalent to 25% of patients being censored in the first year and rising to 57% at the end of 3 years. This number includes patients who will develop AF, for example, detected by routine ECG during follow-up visits or by ECG monitoring initiated at the study sites. Taking this into account, the number of patients per group increases to 1,700. To account for slight variation of trial duration and variability between estimated and observed event rates, the trial protocol includes an interim analysis of the combined enrolment and event rates (blind to random group), with the possibility to adapt sample size.

## Conclusion

In summary, the optimal antithrombotic therapy in patients with AHRE but without diagnosed AF cannot be derived from existing data. The information gained from NOAH–AFNET 6 and from the ARTESiA trial (NCT01938248) will provide sound evidence to guide the use of oral anticoagulation in patients with device-detected AHRE and possibly in patients with atrial arrhythmias documented by other devices. The results have the potential to inform future guidance on the management of patients with atrial arrhythmias detected by implantable devices.
